# Heart rate variability interventions for concussion and rehabilitation

**DOI:** 10.3389/fpsyg.2014.00890

**Published:** 2014-08-13

**Authors:** Robert L. Conder, Alanna A. Conder

**Affiliations:** ^1^Department of Sports Neuropsychology, Carolina Neuropsychological ServiceRaleigh, NC, USA; ^2^Pediatric and Sports Neuropsychology, Carolina Neuropsychological ServiceRaleigh, NC, USA

**Keywords:** heart rate variability, concussion, mild TBI, biofeedback, neurofeedback, rehabilitation

## Abstract

The study of heart rate variability (HRV) has emerged as an essential component of cardiovascular health, as well as a physiological mechanism by which one can increase the interactive communication between the cardiac and the neurocognitive systems (i.e., the body and the brain). It is well-established that lack of HRV implies cardiopathology, morbidity, reduced quality-of-life, and precipitous mortality. On the positive, optimal HRV has been associated with good cardiovascular health, autonomic nervous system (ANS) control, emotional regulation, and enhanced neurocognitive processing. In addition to health benefits, optimal HRV has been shown to improve neurocognitive performance by enhancing focus, visual acuity and readiness, and by promoting emotional regulation needed for peak performance. In concussed athletes and soldiers, concussions not only alter brain connectivity, but also alter cardiac functioning and impair cardiovascular performance upon exertion. Altered sympathetic and parasympathetic balance in the ANS has been postulated as a critical factor in refractory post concussive syndrome (PCS). This article will review both the pathological aspects of reduced HRV on athletic performance, as well as the cardiovascular and cerebrovascular components of concussion and PCS. Additionally, this article will review interventions with HRV biofeedback (HRV BFB) training as a promising and underutilized treatment for sports and military-related concussion. Finally, this article will review research and promising case studies pertaining to use of HRV BFB for enhancement of cognition and performance, with applicability to concussion rehabilitation.

Heart rate variability (HRV) has emerged as an essential component for study, research and clinical applications in physiology and psychophysiology (Moss et al., 2013). Research has shown implications for HRV in physiology, pathophysiology, psychology, psychopathology, cognition, and neurocognitive impairment (Gevirtz, 2013). Within the physical domain, HRV has been postulated as a measure of not only cardiac health, but of cardiac pathology and as a marker of possible mortality from cardiopathology (Bigger et al., 1995). Psychologically, given its connections with the autonomic nervous system (ANS) and limbic system, HRV can be a marker for anxiety disorders, such as a generalized anxiety disorder (GAD), post-traumatic stress disorder (PTSD), or a general predisposition to react sympathetically to external or internal stressors (Blechert et al., 2007). Cognitively, persons with high HRV have shown superior performance on neurocognitive measures of attention, concentration, working memory, and executive functioning (Hansen et al., 2003). Alternatively, HRV can be adversely affected by concussions or any degree of traumatic brain injury (TBI; Goldstein et al., 1998). However, there is research that HRV and HRV impairment can be modified and trained through exercise (Hedelin et al., 2001; Hansen et al., 2004; Hautala et al., 2009), diet (Lima-Silva et al., 2010), and biofeedback interventions (Lehrer et al., 2013). This article will first review theoretical models of the heart–brain ANS interaction, with specific emphasis on the adverse effects of sport and military concussions on HRV. Second, this article will review studies suggesting that heart rate variability biofeedback (HRV BFB) is a promising intervention for treatment of sport and military concussions.

## EPIDEMIOLOGY AND DIAGNOSIS OF TRAUMATIC BRAIN INJURY

First, it is necessary to review the etiology and severity of TBI to understand where concussions fall along the continuum of severity of injury and how they may affect HRV. TBI is defined as an injury to the brain resulting from blunt trauma to the head or body, or acceleration or deceleration forces transmitted to the brain (Barr and McCrae, 2011). TBI severity is classified as Mild, Moderate, or Severe based on degree of injury severity, including presence and length of loss of consciousness (LOC) and number and degree of post-traumatic symptoms. These three categories are based on assessment with the Glasgow Coma Score (GCS; Teasdale and Jeanette, 1974). The GCS measures a patient’s level of functioning based on eye opening and verbal and motor responses, resulting in a score ranging between 3 (minimum) and 15 (maximum). The Mild GCS group has a score of 13–15, and typically will have minimal or no permanent neurologic sequelae, with typical recovery expected in 1–3 months (Levin et al., 2012). The Moderate TBI group has a GCS score of 9–12 and may have permanent sequelae impacting personal life, school or work; finally, the Severe TBI group has a GCS score of 8 and below, and will almost always present with permanent neurologic damage at a moderate level or greater, affecting all aspects of life. It is the “Mild” group that is the primary focus of this article, as these are the athletes and soldiers that most often will be seen clinically and who may have cardiac correlates of concussion sufficient to interfere with cognitive and cardiovascular resources needed for athletic and military performance. Generally, patients with a GCS in the 13–15 range will not have LOC, but may have episodic cognitive confusion, transient amnesia, dizziness, slow reaction time, and balance problems (McCrory et al., 2013). When assessed in the Emergency Room, the traditional physical neurologic exam will be negative and non-focal for pathology, as will the Head CT. The traditional Emergency Room concussion evaluation protocol may not elucidate underlying neuropathology, which is being seen in controlled research studies of athletes with sophisticated neuroimaging including magnetic resonance spectroscopy, fMRI, or diffusion tensor imaging (DTI; Bluml and Brooks, 2006; Pardini et al., 2011) or neuroelectrical assessment including EEG and ERP (Broglio et al., 2009; McCrea et al., 2010; Barr et al., 2012). If someone with a presumed Mild TBI does present with greater neuropathology, such as a basilar skull fracture, intracerebral bleed, or cerebral hematoma, then they are generally referred to as a “complicated” Mild TBI and the severity of injury is noted, with implications for a more complicated recovery.

Giza and Hovda (2001) have elucidated an animal neurometabolic model of mild TBI or concussion, in which there is a significant mismatch between glucose metabolism and regional cerebral blood flow, with a concomitant influx of glutamate and other ionic changes in the cell membrane. Their studies have repeatedly shown a return to baseline neurometabolism around seven days post-injury, presumably without permanent cellular damage. These animal studies have provided the basis for predicted return to baseline functioning in athletes in 7–10 days. This neurometabolic model may be useful to explain the quick recovery typical of non-refractory concussions. However, refractory concussions (the focus of this article) may be better explained by a model of neuronal deformation induced by the biomechanical force of the injury (Abolfathi et al., 2009).

McCrory et al. (2013) postulate that concussions may overlap with the lower end of the Mild TBI spectrum, and may extend to a lesser degree of severity labeled Minimal TBI. The terms “concussion” and “Minimal TBI” may be used interchangeably and as an alternative to Mild TBI but “concussion” will be used in this article, consistent with its usage in sports medicine.

Bigler (2008) points out that a concussion from a football tackle, automobile accident or blast injury does not solely affect the brain. The concussive forces can affect all organs, including the heart (Cernak and Noble-Haeusslein, 2009). Cardiac sequelae of a primary blast injury may produce arrhythmias, ischemia or a myocardial infarction (Garner and Brett, 2007), even in the absence of cerebral concussion. For example, comotio cordis is an unfortunate and often fatal cardiac event from a direct blow to the chest wall, usually in baseball, and without a concomitant cerebral concussion. Additionally, Palma and Benarroch (2014) reviewed non-traumatic cerebral illnesses which impair HRV, including epilepsy, ischemic, and hemorrhagic stroke and neurodegenerative diseases, such as Parkinson’s. They postulate that these neuropathologies involve the insula, basal ganglia, and brainstem and may impair HRV, leading to secondary cardiac illness.

While there have been studies measuring actual G-force on the playing field (Guskiewicz and Mihalik, 2011), few such studies have been done for the most common cause of concussion in adults: automobile accidents. In children, the most common cause of concussion presenting at emergency departments is bicycle accidents (Gilchrist et al., 2011), followed by sport-related injuries. The change in momentum (impact) and transfer of kinetic energy in low impact auto accidents can produce a concussion, and these kinetics and V-max are presumed to produce greater forces than those experienced on the playing field.

Other factors that can impact concussion recovery include protective health status and motivation. Athletes and soldiers tend to have greater cardiovascular and aerobic fitness, which has been postulated as a protective factor for recovery from complicated concussion (Kontos et al., 2006), as opposed to the general, sometimes deconditioned, population. Psychosocial factors, such as motivation, also can impact recovery trajectory. Ponsford et al. (2000) tracked emergency room TBI admissions by mechanism of injury (MOI). After three months, the athletes who presented at the emergency room were significantly less symptomatic than those who presented with a motor vehicle accident as their MOI. Data is not currently available on quantification of blast injury for soldiers in theater, or their outcomes regarding concussion. Nevertheless, typical sequelae of a refractory Mild TBI include problems in attention, concentration, working memory, and executive functioning. Even though the MOI is different between blast injuries (Garner and Brett, 2007) and athletic injuries, the neuropsychological outcomes are similar (Belanger et al., 2009; Cooper et al., 2012) and symptom differences may be due to PTSD in the military group (Lippa et al., 2010). Tan et al. (2009) report a complex, synergistic relationship between PTSD, pain, Mild TBI, and HRV in OEF/OIF veterans.

## HEART RATE VARIABILITY

As this article focuses on the relationship between HRV, cognition, and concussion, an explanation of HRV is necessary. While a full explication of HRV assessment and treatment is beyond the scope of this article, for our purposes we are interested in the measures traditionally cited in the research literature that are amenable to measurement and intervention in the clinic and in exploring the cardiac correlates of concussion. HRV is measured as part of the cardiac QRS complex. The Inter-Beat Interval (IBI) measured between R-waves is the basis for measurement of HRV. HRV was initially felt to be more artifacts, but now is itself the focus of intense study (Lehrer, 2013). HRV is also useful in that it can be measured with minimal hardware and software. A traditional EKG with as few as three chest leads or a three-lead wrist placement with electrodes can be used (Thought Technology, Montreal, Canada). A Blood Volume Pulse sensor (photoplethysmograph) attached to the distal phalange of a finger or the earlobe can be used with appropriate hardware and software to also calculate the IBI (HeartMath, Boulder Creek, CA, USA; StressEraser, New York, NY, USA). There are two standard metrics for measuring HRV for analysis and treatment. One is the time domain (changes over time) and the other is the frequency domain (measurement of a spectrum of oscillatory components of the heart). While there are multiple measures of time domain, the most common used statistical method is measuring the Standard Deviation of the Normal-to-Normal interval (SDNN). The SDNN is basically normalization of the standard deviation of the R-to-R interval, with artifacts removed. Comparisons of data should be with equal epochs, usually 5 min for SDNN. The power spectrum measurements are fractionations of all the oscillatory frequencies contained within a specific epoch. The frequencies are generally calculated over the same epoch as the SDNN metric. There are three frequency sub-bands of particular interest within HRV. The very low frequency (VLF) is composed of frequencies less than 0.04 Hz. The low frequency (LF) is composed of frequencies between 0.04 and 0.15 Hz. The High Frequency (HF) band investigates frequencies between 0.15 and 0.4 Hz. There are both physiologic and cognitive correlates of these frequency bands. The VLF band is considered to reflect mainly sympathetic activity. The LF band is considered to show parasympathetic activity, as well as the baroreflex and enhanced cognitive processing. The HF band is considered to reflect respiratory sinus arrhythmia, and activity from the vagus nerve (Combatalade, 2010).

These same hand-held HRV monitoring devices can provide visual and/or auditory feedback to train HRV. HRV training begins with breathing exercises to increase respiratory sinus arrhythmia, and help one find their resonant frequency – the frequency at which greatest HRV occurs. Breathing rates between 4.5 and 6.5 breaths per minute will produce the greatest HRV in most persons. The biofeedback instruments can provide a visual pacer for breathing rates and informs the person when they have achieved the greatest coherence between respiratory rate and heart rate. A more detailed training protocol is reported by Lehrer et al. (2000).

## NEURAL RELATIONSHIP BETWEEN BRAIN AND HEART

Thayer et al. (2009, 2012) and Thayer and Lane (2009) have postulated an intricate Neurovisceral model of the relationship between the prefrontal cortex of the brain and the heart. In the Thayer model, prefrontal brain areas, including the orbitofrontal cortex and the medial prefrontal cortex tonically inhibit the amygdala, with disinhibition of the central nucleus of the amygdala. The deactivation of inhibitory nuclei leads to a net increase in sympathetic activity which eventuates in decreased HRV and increased heart rate. The central nucleus of the amygdala is believed to be the major efferent source of modulation of autonomic, endocrine, and cardiovascular responses. Three routes are postulated. One involves the activation of tonically active sympathoexcitatory neurons of the rostral ventrolateral medulla due to decreased inhibition from neurons in the caudal ventrolateral medulla, resulting in an increase in sympathetic activity. The second involves inhibitory neurons in the nucleus of the solitary tract, which can lead to a decrease in overall parasympathetic activity. The third postulated pathway involves direct excitation of sympathetic rostral ventrolateral medulla neurons, further leading to an increase in sympathetic activity. The overall result, regardless of the pathway, would be an increase in sympathetic output and overall heart rate, with a concomitant decrease in HRV. While these pathways have not been validated either electrophysiologically or with physiologic staining techniques, Lane et al. (2007) used positron emission tomography (PET) to measure medial prefrontal activity along with simultaneous measurement of spectral HRV. In these studies, subjects were shown film clips depicting emotional situations, involving happiness, sadness, or disgust. In all experimental conditions, HF HRV was correlated with activation of the right prefrontal cortex.

Thayer and Lane (2009) also elucidate hemispheric differences in cardiac activation. Aron et al. (2004) postulate that the right prefrontal cortex may have more potent input for cardiac modulation. This is not surprising, given the functional neurology of the right prefrontal cortex involved in emotional regulation and dysregulation. Nevertheless, the models propose that prefrontal activity will modulate cardiac output, including general heart rate and HRV. However, Mild TBI or concussion injuries are rarely lateralized, typically being more diffuse in etiology and MOI.

Williamson et al. (2013) propose a similar model. They propose a model of connections between the orbitofrontal cortex through the uncinate fasciculus to the amygdala, terminating in the sympathetic nervous system. The importance of these white matter tracts for this model is that they communicate from the prefrontal cortices to the brain stem, then to the heart. Disruption of the white matter tracts may induce a loss of inhibitory control upon theANS. Not only does this loss of inhibitory control result in impairment of cognitive abilities, but it also can result in loss of emotional regulation, as seen in soldiers and civilians with PTSD. This review cites similar neuroimaging studies to Thayer et al. (2009) as well as citing neurotransmitter involvement.

Studies using DTI assess the integrity of the white matter tracts, whereas typical MRI including T1, T2, and FLAIR protocols may not show neuropathology in white matter. In DTI studies, fractional anistrophy (FA) and mean diffusivity (MD) are measures of white matter integrity noted to be impaired in concussion. Disruption may result in loss of neural transmission or in reduction in transmission time due to neural deformation from physical trauma (Bazarian et al., 2007).

## MECHANISM OF INJURY IN CONCUSSION

As noted above, children from pre-school to senior high school are most often injured in bicycle accidents. Among teen sports, American football is the next most frequent concussion generator. By gender in this age group, American football is the greatest concussion generator for males, while soccer is the greatest concussion generator for females. Both sports can provide blunt trauma at high velocity, either directly through head-to-head strikes, foot- or knee-to-head strikes, or striking the ground or another object, such as the goal. Due to the jagged cranial cavity, most often the physical injury to the brain impacts the orbitofrontal and anterior temporal areas. Linear forces are more often involved in head-to-head contact of athletes and persons in motor vehicles with straight-line acceleration, then impact, followed by rapid deceleration. In contrast, rotational injuries may be seen in open-field tackles or checking or boarding on the ice of a hockey rink.

While traditional neuroimaging will not show white matter attenuation, a recent study of Division I Ivy League football players with subconcussive injuries (not diagnosed with a formal concussion) revealed changes in FA and MD DTI after a season of play (McAllister et al., 2014). The groups compared contact sports, such as American football and soccer versus non-contact sports, including cross country and track and field. There were significant changes in FA and MD in the contact sport group over the course of a season. Long-term follow up is needed to ascertain if these changes are reversible (some data points to this) or if they are non-reversible. However, this does validate the concerns about the integrity of white matter tracts as being the conduit between the heart and brain.

Thompson and Hagedorn (2012) report alterations in HRV in patients with concussion or mild TBI, including low amplitude and poor rhythmicity. Decrease in HRV has been seen among patients with all levels of TBI severity, from Severe TBI (Baugley et al., 2006) to concussion (Goldstein et al., 1998; La Fountaine et al., 2009). Disruption in cardiovascular reactivity was noted after sports concussion, with interruptions of middle cerebral artery blood velocity after oxidative stress (Len et al., 2011). Len et al. (2011) studied HRV in Canadian Junior Hockey League players with and without concussion and matched for demographic characteristics. In the resting condition, there were no differences noted. However, upon exertion, there were significant differences in total HRV, as well as LF and HF power for the concussed group. This has implications for elite athletes who quickly need to respond with greater cardiovascular output to perform well in their sport (Gall et al., 2004). By extension, this cardiovascular output dysfunction may also hold true for soldiers concussed in IED blasts. Similarly, Leddy et al. (2007) fully reviewed the ANS changes from concussion, including “greater sympathetic and lower parasympathetic activity …” and cerebrovascular dysregulation. These concussive changes may affect multiple organ systems, including pulmonary, hepatic, and renal. They suggest an individualized aerobic exercise program that is below the threshold of symptom onset.

## HRV AND EEG

As multiple models postulate a heart–brain connection in HRV, measurement can empirically validate this neurovisceral relationship. Searches of both Medline and PsychInfo revealed only a few studies that addressed measurement of EEG changes with HRV intervention. Sherlin et al. (2010) measured EEG variables during HRV training. They assessed 19-channel QEEG and sLORETA variables in a group of laboratory stressed-induced (non-injured) subjects undergoing HRV training. Significant changes in either alpha increase or beta decrease were found at Brodman’s areas 24, 30, and 31, all associated with the limbic system and cingulate gyrus. The authors postulate these measured EEG changes from HRV BFB reflect a decrease in autonomic arousal in brain areas critical for stress regulation.

Prinloo et al. (2013) examined EEG correlates of HRV intervention in stressed senior managers, further exposed to experimental laboratory stress. In this study, five EEG sites were monitored. Two frontal sites (Fp1 and Fp2) were monitored for muscular artifacts. The important midline sites of Fz, Cz, and Pz were monitored, as they are thought to reflect brain attention and arousal mechanisms. After a single session of HRV BFB, significant changes were found with reduced beta and increased theta at all three central sites. The authors suggest these EEG changes are reflective of “… increased relaxation, decreased anxiety and decreased mental effort … (p. 31).” These EEG changes were associated with increases in LF and SDNN HRV variables.

Reid et al. (2013) assessed EEG changes in 40 clinical subjects, half of whom were athletes undergoing optimal performance training, including HRV BFB. For this study, only one EEG site was utilized: Cz, the primary central site of the vertex, reflecting the sensorimotor rhythm (SMR) at 12–15 Hz. Clients showing successful HRV training (peak frequency heart rate between 0.05 and 0.15 Hz) also showed significant increases in SMR amplitude. The authors conclude that the SMR increase reflects a state of relaxed anticipatory focus (Sterman, 1966) useful for athletes who need to perform better in stressful sport competitions, with greater flexibility and regulation over their ANS. By extension, soldiers might also benefit from this approach.

Collura (2009) developed a software and hardware protocol (BrainMaster, Bedford, OH, USA) that combined HRV and Alpha EEG training. Each parameter could be trained independently or simultaneously for greater HRV and Alpha enhancement. Bazanova et al. (2013) trained high Alpha EEG (10–12 Hz) to measure its effect on HRV. They found that healthy male subjects with low resting levels of Alpha who increased Alpha had lowered EMG, greater HRV and showed increases in cognitive performance. They also noted that these changes were not present in a control group that did not receive any feedback, thus validating the need for actual feedback for self-regulation.

To date, only case studies have been conducted on concussed patients using either HRV or Neurofeedback interventions. Lagos et al. (2013) used a 10-week HRV BFB protocol on an adult athlete with post-concussion syndrome. At the end of treatment, the patient showed greater HRV, and greater LF power, as well as significant reduction in severity of post-concussion headaches and overall post-concussive symptoms. Thompson et al. (2013) report the case of an athlete who received a sport-related concussion and was treated with a multi-modal intervention, including both HRV BFB and neurofeedback. Bhandari et al. (2013) used both HRV and neurofeedback to treat an adult male with a Severe TBI. While this patient was much more severely injured than a person with a concussion, after a lengthy course of treatment he showed improvement on multiple QEEG parameters, as well as three HRV parameters, and was able to function successfully in his personal and work life. Lagos et al. (2012) provide a comprehensive rationale for HRV BFB in prolonged PCS from hyperactivation of the sympathetic nervous system and hypoactivation of the parasympathetic nervous system. In addition to changes in HRV as an outcome measure for BFB intervention, they also recommend multimodal measures, such as cardiovascular and neurovegetative functioning, and quality of life indicators.

## NEUROCOGNITIVE ENHANCEMENT AND REHABILITATION WITH HRV BFB

Several compelling studies have implicated HRV BFB in neurocognitive enhancement, particularly executive functioning and working memory. In studying non-injured persons, Hansen et al. (2003) divided military personnel on the basis of low or high HRV groups and then assessed sustained attention and working memory. As would be expected, the higher HRV group had superior performance on both measures. Other studies of executive functioning, including the ability to make decisions, plan, and benefit from feedback, have linked executive skills to high HRV. Murray and Russoniello (2012) divided university students between exercise or control groups. HRV including instantaneous changes of heart rate and spectral analysis of HRV were measured. Results indicated optimal performance with the peak inverted-U curve for both the complex Trail Making B task and a four-choice reaction timed complex test. Optimal neurocognitive performance was obtained in the LF band. These studies suggest that HRV training may be a viable intervention to promote executive function rehabilitation in PCS.

Collegiate basketball players with high-state anxiety levels were given ten minutes of HRV training for ten days to increase coherence and respiratory sinus arrhythmia (Paul and Garg, 2012). The premorbidly anxious collegiate basketball players showed improvement on objective measures of dribbling, passing, and shooting with increases in HRV, especially the LF range. This study has implications for HRV BFB training in the rehabilitation of autonomic and emotional dysregulation as a critical component in PCS recovery. In a study with military relevance (Saus et al., 2006), Norwegian Police Academy cadets with higher HRV were noted to have greater situational awareness, and to perform more accurately in complex shooting drills requiring focused attention and executive abilities. A compelling study not yet undertaken would be to investigate whether pre-deployment training in HRV could possibly reduce anxiety-related aspects of combat, including PTSD.

## SUMMARY AND CONCLUSION

In summary, this article has attempted to elucidate the relationship between the brain, particularly the prefrontal cortices, the ANS, and the heart. There can be top-down or bottom-up interaction to both, such that the prefrontal cortices’ judgment of ambiguity for threat attenuates HRV. Similarly, baseline higher levels of HRV, especially LF HRV, are associated with greater performance on complex neurocognitive tasks of concentration, working memory, and executive functioning, requiring prefrontal integrity. Further, as sports-related and perhaps military-related concussions interfere with optimal levels of HRV, interventions to restore or increase these optimal HRV levels are needed. Diet, endurance/cardiovascular exercise, and biofeedback are effective interventions. Of these three, HRV BFB can be accomplished with minimal hardware, software, time, cost, and effort constraints upon an individual over a period of a few days. Additionally, biofeedback provides real-time feedback for the attribute of interest, allowing the athlete or soldier to exercise cognitive control over their physiology.

Future research is needed to assess the EEG correlates of HRV intervention on randomized, controlled groups of symptomatic post-concussion athletes and soldiers. As Tan et al. (2013) noted, HRV intervention can be more acceptable for veterans with PTSD than the emotionally charged psychotherapies, and this same logic may hold true for athletes and soldiers with PCS. Data currently available regarding HRV BFB efficacy raise a compelling argument for the need for further empirical validation, not only for treatment of refractory sport and military concussions, but possibly pre-deployment stress inoculation training for soldiers, and pre-game training for athletes.

## Conflict of Interest Statement

The authors declare that the research was conducted in the absence of any commercial or financial relationships that could be construed as a potential conflict of interest.
